# Effects of Organic Vehicle on the Rheological and Screen-Printing Characteristics of Silver Paste for LTCC Thick Film Electrodes

**DOI:** 10.3390/ma15051953

**Published:** 2022-03-06

**Authors:** Yujun Gao, Jingjing Feng, Feng Liu, Zhifu Liu

**Affiliations:** 1 CAS Key Lab of Inorganic Functional Materials and Devices, Shanghai Institute of Ceramics, Chinese Academy of Sciences, Shanghai 200050, China; gaoyujun@student.sic.ac.cn (Y.G.); liuf@mail.sic.ac.cn (F.L.); 2 Center of Materials Sciences and Optoelectronics Engineering, University of Chinese Academy of Sciences, Beijing 100049, China

**Keywords:** LTCC, silver paste, organic vehicle, rheological behaviors, fine-line screen-printing

## Abstract

Silver paste is widely used for low-temperature co-fired ceramic (LTCC) electrodes. In this work, a kind of LTCC silver paste for fine-line screen-printing was developed by considering the effect of the organic vehicle on rheological behavior and screen-printing properties. A step-by-step volatilization mode was applied to screen the mixed organic solvent of *α*-terpineol, 2-(2-butoxyethoxy) ethyl acetate (BCA) and dibutyl phthalate (DBP). The *α*-terpineol:BCA:DBP ratio of 5:2:3 is selected by considering the volatility, viscosity, and pseudoplasticity of the organic vehicle. Both viscosity and pseudoplasticity of shear-thinning increase with the increase of ethyl cellulose (EC) organic binder content. Three interval thixotropy test (3ITT) was conducted to discuss the thixotropy of silver paste. The minimum printing line width of 13.27 µm is obtained using silver paste with 10 wt% EC, confirming that the homemade paste has good printability.

## 1. Introduction

Low-temperature co-fired ceramic (LTCC) technology is considered to be an important approach for high-frequency applications due to the advantages in compact packaging and good signal transmission for 5G/6G mobile communication systems [1,2,3]. The conductor thick film compositions used in LTCC systems must be chemically compatible with green ceramic tapes during the sintering process, except for excellent electrical performance [4,5]. Silver is the preferable conducting material in LTCC applications, owing to its excellent compatibility, superior electrical conductivity, and low sintering temperature [6,7,8]. Silver paste usually consists of silver powder, inorganic binder, and organic vehicle [9,10,11,12]. Inorganic binder promotes the sintering of silver powder and the bonding between silver electrode and LTCC substrate. An organic vehicle is an organic mixture of polymer binder, solvent, etc., which disperse the above powders to impart desired rheological properties [13,14,15]. Therefore, the rheological behavior of silver pastes is mainly controlled by the organic vehicle, and largely affects its printing performance.

An organic vehicle is required to have different volatilization rates to guarantee the leveling and storage stability of silver paste in different stages [16,17]. If the volatilization rate of organic solvent is too fast, the organic vehicle and silver paste cannot not be stored for a long time. In contrast, the low volatility of organic solvents is not good for the drying of silver paste and for obtaining fine patterns. Therefore, the mixed solution with low volatility at room temperature and high volatility at high temperature was preferred for the solvent of an organic vehicle [18]. The most commonly used solvents in an organic vehicle are ester alcohols and terpenes, such as terpineol or their mixtures with other solvents, and the slight change of solvent content will have a great influence on the rheological properties of silver paste [19]. Various combinations of these solvents are needed to obtain the required volatility.

On the other hand, the organic vehicle (especially organic binder) plays a key role in controlling the rheological behaviors and realize high-resolution screen-printing [20,21,22]. High-quality paste should have optimum shear-thinning pseudoplasticity, allowing paste flow in screen mesh, and have a suitable thixotropy for paste structure recovery so as to ensure the shape of the printed pattern [23,24,25,26]. For example, the shear-thinning behavior becomes significant with the increase of EC (ethyl cellulose) molecular weight [14]. In order to meet the development trend of miniaturization of electronic components, the width of screen-printed fine lines needs to have higher precision. Therefore, it is very necessary to systematically study the effects of an organic vehicle, especially the solvent composition and EC content in the mixed solvent, on the rheological behavior and fine-line screen-printing characteristics of silver paste.

Hence, a kind of LTCC silver paste for fine-line screen-printing was developed by optimizing the composition of an organic vehicle, including solvent and binder. A step-by-step volatilization mode was applied to screen the mixed organic solvent of *α*-terpineol, BCA, and DBP for organic vehicles. Then, the solvent ratio was determined by studying the effects of mixed solvents on volatility, viscosity, and pseudoplasticity of organic vehicle. The effect of EC content on viscosity and pseudoplasticity of silver paste for LTCC applications was studied. Screen-printing characteristics and thixotropy of silver paste were conducted to optimize the composition of organic vehicle.

## 2. Experiments

*α*-terpineol (A), BCA (B), and DBP (C) were selected as organic solvents, and their composition can be written as A*_x_* B*_y_* C*_z_*, where *x*, *y*, and *z* indicate the amount of each component. The mixed solvent was firstly weighed and mixed according to the ratios (*x* = 2, 3, 4, 5, 6, 7; *y* = 2, 3, 4, 5, 6, 7; *z* = 1, 2, 3) in Table 1 to explore the volatility characteristics of the mixture at 60 °C, 90 °C, 120 °C, 150 °C, 180 °C, and 210 °C for 20 min. The experiment was repeated more than three times and the average experimental results were calculated to obtain the solvent volatilization loss. Then, a certain amount of EC (Shanghai Aladdin Biochemical Technology Co., Ltd., Shanghai, China) was added to the optimized solvent. The mixture was stirred at 85 °C for 2 h to dissolve EC to obtain the organic vehicle.

Silver powder used in this study was supplied by Guangdong FENGHUA Advanced Technology Holding Co., Ltd., Zhaoqing, China, with particle size in the range of 1–3 μm. Homemade Ca-B-Si-O glass frit was used as an inorganic binder. The Ca-B-Si-O glass frit was prepared by the melt-quenching method. Uniform stoichiometric mixtures of oxide powders were mixed in a mortar and melted in an elevator furnace at 1500 °C for 1 h. The melt was then quenched in de-ionized water and ground to 1–2 μm glass powder in a sand-milling machine. Silver powders (82 wt%), organic vehicle (16 wt%), and glass frits (2 wt%) were poured into a 40 g plastic jar and mixing them for 30 min using a mixing machine (SINO, VM300S, Mianyang, China). Subsequently, the mixture was milled for 45 min using a three-roll mill (Zili, SDT50, Changzhou, China) to eliminate agglomerates in the paste. Screen-printing tests were carried out by printing silver paste on LTCC green tape using a screen-printing machine. After leveling for about 10 min at room temperature, the screen-printed thick film was dried for 3 min in an oven at 100 °C.

The rheological properties of the formulated silver paste were investigated using a rotational rheometer (Anton Par, MCR 302, Graz, Austria) equipped with a 25 mm-diameter lower plate and 2° upper cone. The gap distance was equal to 1.0 mm, and all measurements were probed at 25 °C. Silver paste need to stand for 3 min before each test to minimize the memory effects from the previous loading procedure. The steady-shear flow experiment was performed at shear rates of 0.1–1000 s^−1^ to measure the shear viscosity, and the obtained viscosity data were used to plot the viscosity curve as a function of shear rate. 3ITT (three interval thixotropy test) with constant shear strain were performed to simulate the paste behavior during the screen-printing process. The first interval is related to the paste on screen meshes before screen-printing, and a shear strain was fixed at 0.5% for 80 s, and the frequency was 1 Hz. Then a shear strain of 500% was applied for 28 s to reproduce the squeegee stroke during screen-printing. In the third interval, the shear strain is then returned to its initial value (0.5% shear strain) for 120 s and simulates the structure recovering of silver paste. Optical microscope (Leica, DM 2500M, Wetzlar, Germany) and Scanning Electron Microscope (Phenom Pro, Phenom-world, Eindhoven, The Netherlands) were used to observe and measure the widths and micro morphologies of screen-printed lines.

## 3. Results and Discussion

### 3.1. Influence of Solvents on Organic Vehicle

The preparation of LTCC silver paste requires different volatilization rates of organic vehicles in different stages to ensure the leveling and storage stability of the paste [16,17]. It is also necessary to ensure the viscosity and pseudoplasticity of an organic vehicle to adjust the rheological properties of paste and realize high-resolution printing. Therefore, this section studies the effects of mixed solvents on volatility, viscosity, and pseudoplasticity of organic vehicle.

**Figure 1:**
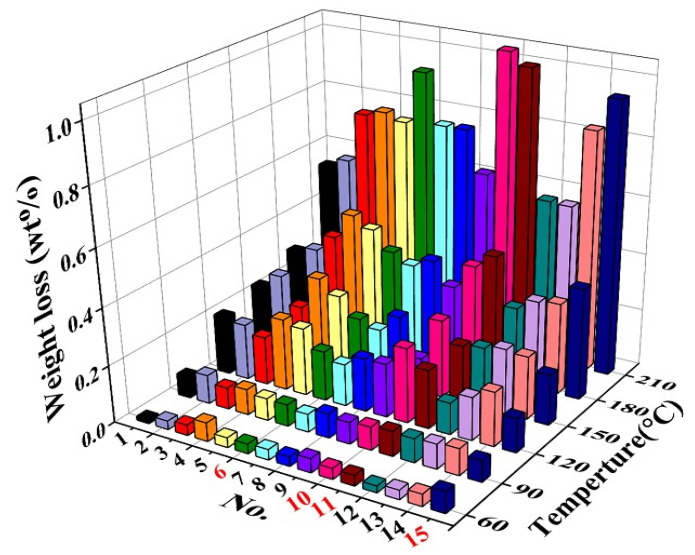
The volatilization characteristics of organic solvents at different temperatures.

Considering the volatility of a single solvent is proportional to its boiling point [27], and the volatile characteristics can be controlled by changing the component ratio of mixed solvents according to Henry’s law [28], we studied the volatile properties of the mixed solvents of *α*-terpineol, BCA, and DBP, which have boiling points of 214.0–224.0 °C, 245 °C, and 340 °C, respectively. As shown in Table 1, fifteen groups of mixed solvents with different proportions of terpineol, BCA, and DBP were designed. The volatility change of mixed solvents with different proportions is shown in Figure 1. The volatility of mixed solvents is relatively low at temperatures from 60 °C to 90 °C, which should be attributed to their much higher boiling points than this temperature range. With the increase of temperature, the volatilization of organic solvents increases, and the volatilization of each solvent is the largest at 210 °C. No.6, No.10, No.11, and No.15 exhibit the highest volatilization at 210 °C, which may be due to the higher content of *α*-terpineol whose boiling point is close to 210 °C. Since the low volatility below 100 °C is helpful for the stable storage of paste, and the high volatility between 100 °C and 200 °C is a benefit to the rapid volatilization of solvents after screen-printing, the preferred proportions of *α*-terpineol, BCA, and DBP in the mixed solvent are selected as (7:2:1), (5:3:2), (6:2:2), and (5:2:3), which are used for further investigation.

**Figure 2:**
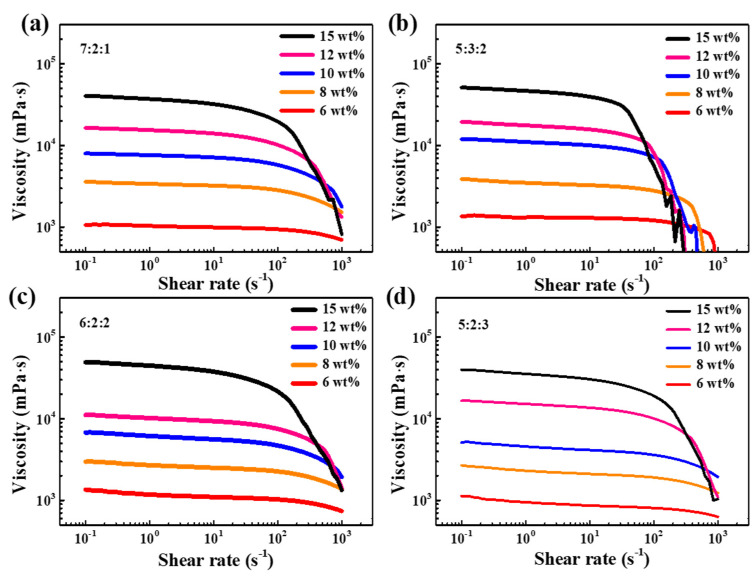
Viscosity of organic vehicles as functions of EC content and proportion of solvents: (**a**) A:B:C = 7:2:1, (**b**) A:B:C = 5:3:2, (**c**) A:B:C = 6:2:2, (**d**) A:B:C = 5:2:3.

**Figure 3:**
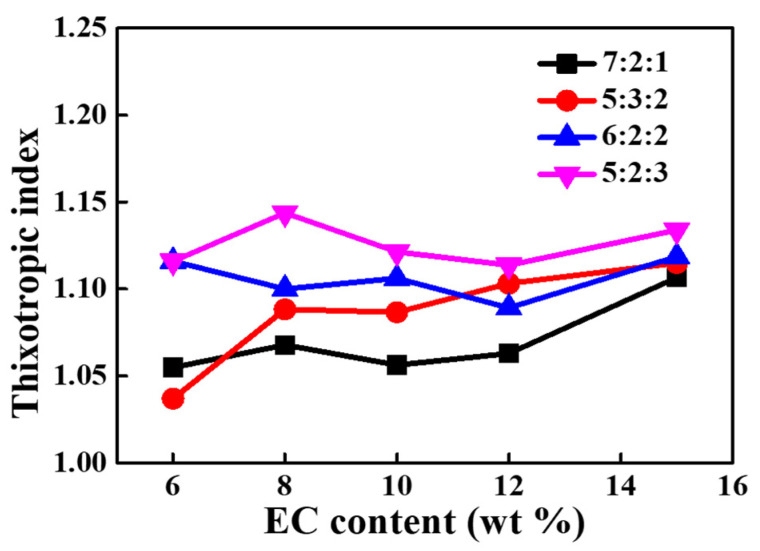
The relationship of the organic vehicle thixotropic index with EC content and the proportion of solvents.

The viscosity-shear rate analysis of organic vehicles with different EC content and solvent ratio are shown in Figure 2. All pastes show good pseudoplastic behavior of shear-thinning. Besides, the viscosities of organic vehicles obviously increase with the increase of EC content, and the viscosity of organic vehicles with different solvent ratios is almost the same. As an important rheological parameter to assess pseudoplasticity, thixotropy index is usually defined as the ratio of viscosity measured at low shear rate such as 0.2 s^−1^ to that measured at a high shear rate such as 2 s^−1^ [29]. As shown in Figure 3, the thixotropy index of organic vehicles with a solvent ratio of 5:2:3 is higher than others. This means organic vehicles at this ratio have better shear-thinning behavior, which is conducive for optimizing the rheological properties of silver paste and realizing high-precision printing.

**Figure 4:**
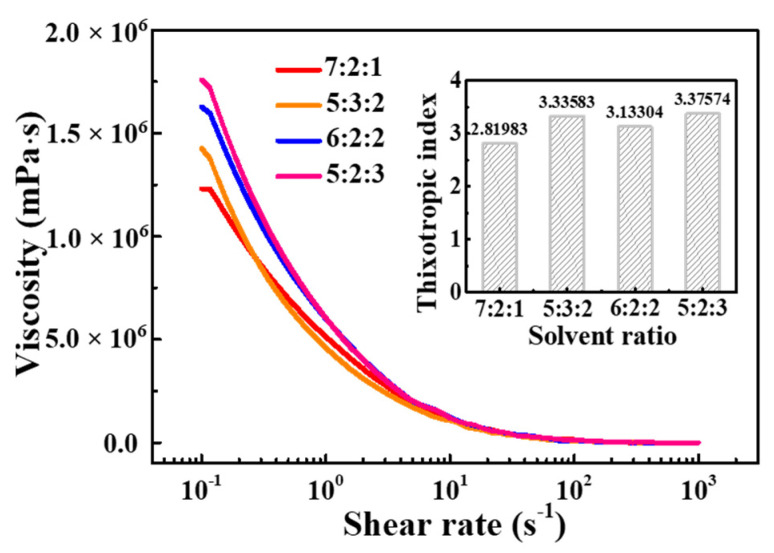
The effect of the proportion of solvents on the viscosity of silver paste with 10 wt% EC content, inset is the thixotropic index of silver paste for different solvent ratios.

To confirm the optimizing composition of the solvent for silver paste, the rheology and printability of the silver paste prepared using the above organic vehicles were studied. As shown in Figure 4, the viscosity changes with the decrease of the shear rate. The paste has great resistance to deformation and shows a relatively high viscosity value at a low shear rate. With the increase of shear rate, the silver powder in the paste easily moves along the shear direction, and EC molecules in the organic vehicle are separated, stretched, and oriented, thus reducing the shear viscosity. The viscosity decreases to the minimum when the shear rate is close to 1000 s^−1^. The thixotropic parameters of those pastes were evaluated and illustrated in the inset of Figure 4. They are 2.820, 3.336, 3.133, and 3.376 for pastes with solvent ratio of (7:2:1), (5:3:2), (6:2:2), and (5:2:3), respectively. Those different thixotropic parameters may attribute to the different inner network strength formed by the intermolecular force, especially hydrogen bond between powders and binders with different solvent ratios [29,30,31]. In particular, the thixotropic parameter of paste with the solvent ratio of 5:2:3 is the largest, which further proves that the solvent with *α*-terpineol:BCA:DBP = 5:2:3 ratio is more suitable for the preparation of silver paste for screen-printing.

### 3.2. Influence of Binder on Silver Paste

**Figure 5:**
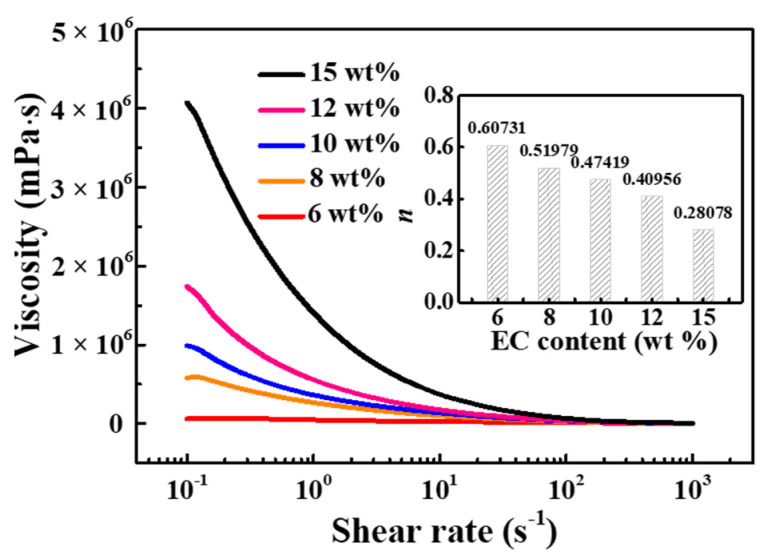
The viscosity curves of silver pastes with different EC content in othe rganic vehicle using 5:2:3 solvent, inset is the shear-thinning constant *n* of silver paste for different EC content.

To satisfy the application of screen-printing, the effects of EC organic binder content on the viscosity and pseudoplasticity of silver paste for LTCC applications were further studied. As shown in Figure 5, the viscosity curves have distinctive variations for silver pastes with different EC content in organic vehicle. The viscosities of silver pastes increase with the increase of EC content, indicating an enhancing interaction between particles of the three-dimensional cross-linked network formed in the paste. Those pastes with different EC content all exhibit good shear-thinning behavior over a shear rate range of 0.1–1000 s^−1^. To have a better understanding of the pseudoplastic behavior of silver pastes, the viscosity-shear rate curves were plotted in logarithmic form based on the Ostwald–de Wale power-law equation:

*τ* = *K* ×*γ ^n^* (1)

*η* = *τ*/*γ* = *K* × *γ ^n−^*^1^ (2)

where *τ* is the shear stress (Pa), *K* is a constant, *γ* is the shear rate (s^−1^), *η* is the viscosity of silver paste, *n* is the shear-thinning constant (*n* < 1). A lower *n* value means the viscosity changes quickly with shear rate, and the pseudoplasticity of silver paste is better. As illustrated in the inset of Figure 5, with the increase of EC content, the *n* value gradually decreased and the pseudoplasticity gradually enhanced, implying an increasing shear-thinning pseudoplasticity.

### 3.3. Screen-Printing Characteristics

**Figure 6:**
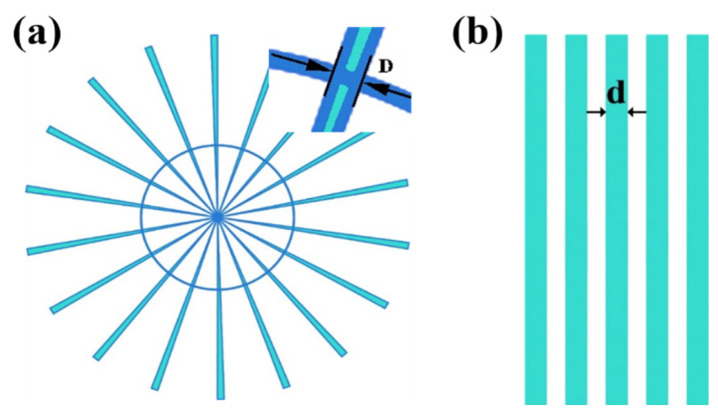
Layout of the screen-printing pattern: (**a**) Siemens star, (**b**) line/space pattern.

In order to determine the best silver paste composition for fine-line screen-printing, a semi-automatic screen printer was used to print patterns shown in Figure 6 on LTCC green tape. The so-called Siemens star displayed in Figure 6a consists of a circle with 18 printed circular sectors, and its linewidth decreases from 870 µm to infinity from edge to center. The test pattern used to evaluate the fine-line resolution also includes a parallel 50/50 μm line/space pattern (Figure 6b). To determine the screen-printing resolutions, the outer interruption was determined, starting from the center of Siemens star. The resulting minimum line width is defined as *D*, and the width of fine line is named *d*.

**Figure 7:**
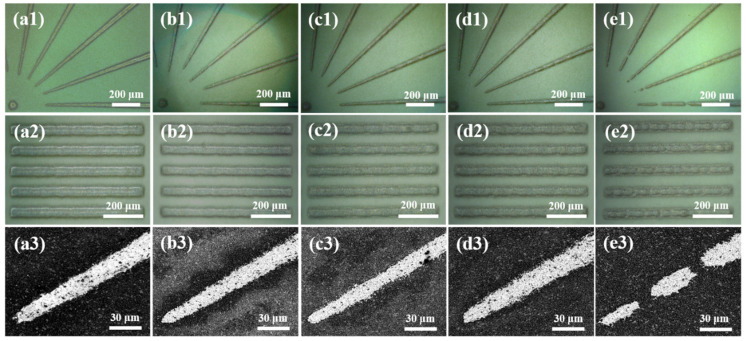
Optical morphologies and SEM images of screen-printed patterns for silver pastes with different EC content: (**a1**–**e1**) optical morphologies of screen-printed Siemens star patterns for silver pastes with 6 wt%、8 wt%、10 wt%、12 wt%、15 wt% EC content; (**a2**–**e2**) optical morphologies of screen-printed line/space patterns for silver pastes with 6 wt%、8 wt%、10 wt%、12 wt%、15 wt% EC content; (**a3**–**e3**) SEM images of screen-printed Siemens star patterns for silver pastes with 6 wt%、8 wt%、10 wt%、12 wt%、15 wt% EC content.

**Figure 8:**
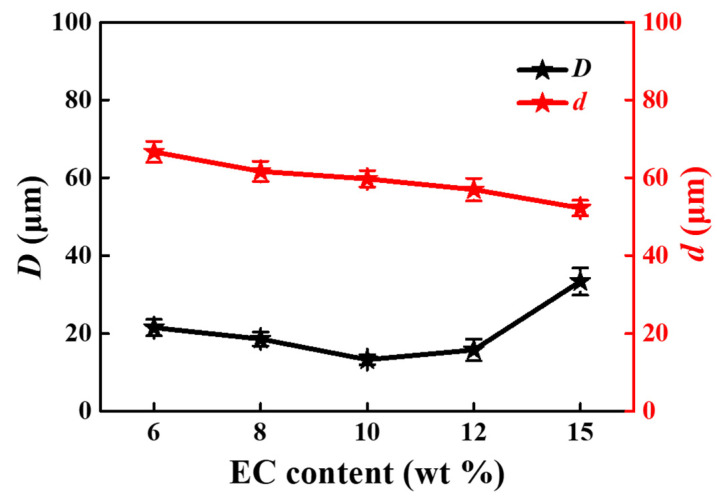
The screen-printing resolutions *D* (black labels) and *d* (red labels) of silver pastes as a function of EC content.

The optical morphologies and SEM images of screen-printed patterns are displayed in Figure 7, which are the screen-printed patterns for silver pastes with EC content of 6 wt% (Figure 7a), 8 wt% (Figure 7b), 10 wt% (Figure 7c), 12 wt% (Figure 7d), and 15 wt% (Figure 7e), respectively. Besides, the specific printing resolution values are summarized in Figure 8. The higher the EC content, the lower line width was obtained. It can be noted here that the edge of screen-printed fine lines at lower EC content seems to expand because the viscosity is low, and a certain flow occurs. However, silver paste with higher EC content showed obvious net marks (in Figure 7d,e), especially for the screen-printed patterns in Figure 7e, which might be due to the results of insufficient leveling time. The screen-printed fine lines in Figure 7 have smooth line edges between the composition of 6–12 wt% EC, while in the case of 15 wt% EC, fine-line patterns are broken in many places because the viscosity is too high, and the line resolution of the test pattern was degraded. It can be seen from Figure 7a3–e3 and Figure 8 that the variation of minimum line width first decreases and then increases with the increase of EC content. Silver paste with 10 wt% EC obtained minimum line width of 13.27 µm, which is superior to the reported values shown in Table 2 [18,19,32,33], indicating that 10 wt% of EC should be the optimum composition.

### 3.4. Thixotropy of Silver Paste

**Figure 9:**
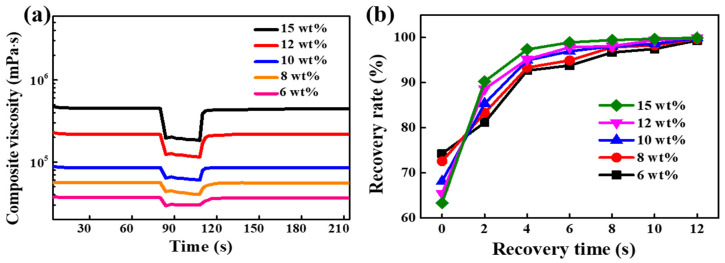
3ITT analysis of silver pastes with different EC content: (**a**) 3ITT curves, (**b**) recovery rate.

To further explain the screen-printing performance of silver pastes, 3ITT was carried out to analyze the dynamic changes in structure and viscosity to understand the thixotropic behavior of silver pastes. As shown in Figure 9a, the complex viscosity of silver paste in the low shear region is generally higher, which is conducive to the stable existence of silver paste on the screen without flowing and dripping before screen-printing. Then, the viscosity in the high strain region decreases sharply, meaning the inner networks damage rapidly, which is beneficial to screen-printing and leveling of the paste. Subsequently, the viscosity gradually recovers in the recovery stage, signifying the reconstruction of silver paste structure. Finally, the relatively high viscosity makes the paste not overflow all around after screen-printing, forming screen-printing pictures and texts, and ensuring dimensional accuracy. Besides, the recovery rate of silver pastes with different EC content is shown in Figure 9b. The recovery rate increases with the increase of EC content in a short time after severe shearing, indicating the structure reconstruction time decrease with the increase of EC content. This further explains the above result that the width of fine line decreases with the increase of EC content.

## 4. Conclusions

A kind of LTCC silver paste for fine-line screen-printing was developed. Considering the influence of solvent ratio on volatility, viscosity, and pseudoplasticity of organic vehicle, the mixed organic solvent was selected. The thixotropic parameter of the organic vehicle with solvent ratio of 5:2:3 is the largest, which is suitable for the preparation of silver paste for screen-printing. Both viscosity and pseudoplasticity of shear-thinning increase with the increase of EC content. The minimum line width of 13.27 µm and good line quality were obtained from silver paste with 10 wt% EC, which was concluded to be the optimum amount. 3ITT finally explains the excellent thixotropy and good printability of homemade paste.

## Figures and Tables

**Table 1:** The ratio of mixed solvents.

No.	Sample	Solvent Ratio	No.	Sample	Solvent Ratio	No.	Sample	Solvent Ratio
1	A_2_B_7_C_1_	2:7:1	7	A_2_B_6_C_2_	2:6:2	12	A_2_B_5_C_3_	2:5:3
2	A_3_B_6_C_1_	3:6:1	8	A_3_B_5_C_2_	3:5:2	13	A_3_B_4_C_3_	3:4:3
3	A_4_B_5_C_1_	4:5:1	9	A_4_B_4_C_2_	4:4:2	14	A_4_B_3_C_3_	4:3:3
4	A_5_B_4_C_1_	5:4:1	10	A_5_B_3_C_2_	5:3:2	15	A_5_B_2_C_3_	5:2:3
5	A_6_B_3_C_1_	6:3:1	11	A_6_B_2_C_2_	6:2:2			
6	A_7_B_2_C_1_	7:2:1						

**Table 2:** Comparison of this work with the state-of-the-art printed fine lines.

Printing Method	Materials	Binder	Fine Line Width	Ref.
3D Printing	Ag	EC	19 μm	[18]
Screen printing	Ag	EC	25 μm	[19]
Screen printing	Ag	EC	44 μm	[32]
Screen printing	Carbon Black, Ag	Resin	100 μm	[33]
Screen printing	Ag	EC	13.27 μm	Our work

## Data Availability

We choose to exclude this statement.
